# Infection cycle of maize stalk rot and ear rot caused by *Fusarium verticillioides*

**DOI:** 10.1371/journal.pone.0201588

**Published:** 2018-07-31

**Authors:** Xiaotong Gai, Huaiyu Dong, Suna Wang, Bo Liu, Zhaoran Zhang, Xiaoyang Li, Zenggui Gao

**Affiliations:** 1 Ministry of Agriculture Key Laboratory of Northern Crop Immunology, College of Plant Protection, Shenyang Agricultural University, Shenyang, China; 2 Institute of Plant Protection, Academy of Agricultural Sciences of Liaoning, Shenyang, China; 3 College of Landscape and Ecological Engineering, Hebei University of Engineering, Handan, China; Universita degli Studi di Pisa, ITALY

## Abstract

*Fusarium verticillioides*, an important maize pathogen produces fumonisins and causes stalk and ear rot; thus, we are aimed to clarify its infection cycle by assessing enhanced green fluorescent protein (*EGFP*) expression in stalk and ear rot strains. Maize seeds were inoculated with stable and strongly pathogenic transformants. To investigate the degree of infection, inoculated plants were observed under a stereo fluorescence microscope, and affected tissue strains were detected using PCR. We found that both transformants infected maize. Hyphae infected the plants from radical to the stem and extended to the ear and infected ear kernels caused a second infection. This process formed the infection cycle.

## Introduction

*Fusarium verticillioides* (teleomorph *Gibberella moniliformis*) is one of the most common causal agents of diseases in maize (*Zea mays* L.) and a wide range of crops and plants worldwide [1]. This fungus produces fumonisins, a group of mycotoxins that can accumulate in the kernels making them toxic to animals and humans [2–7]. In addition to their negative health impacts, studies have revealed that the fumonisins produced by *F*. *verticillioides* cause significant yield losses [8] and also threaten the health of humans and animals [9–11].

Maize is particularly susceptible to *F*. *verticillioides* infection due to the large amounts of fumonisins produced [12, 13].

*Fusarium* stalk rot reduces output in maize by 10% typically and by 30–50% in severely affected areas [14], while *Fusarium* ear rot, characterized by discolored and a reduced number of grains, not only reduces yield, but also influences the quality of the seeds.

As the policy of straw returning (i.e., leaving straw in the field instead of burning to protect the environment) becomes more common in China, maize stalk and ear rot are spreading because *F*. *verticillioides* can survive in crop residues, which initiates subsequent infection [15, 16]. Thus, these diseases have become widespread in maize production, which is significantly influencing yield and causing increasing concern.

Green fluorescent protein (GFP) discovered in *Aequorea victoria* has the advantages of being stable, convenient to use, and easily observed in living cells [17]. Thus, GFP has been used in many studies, including those on the filamentous fungi of *Fusarium*. For example, Ronald and Thomas (2004) used a strain of *F*. *graminearum* transformed with GFP to investigate growth in barley spike and the patterns of seed infections [18]. Additionally, Li et al. (2010) created transformants with GFP to investigate growth in wheat and the pattern of seed infection [19], Qi et al. (2014) evaluated wheat cultivars using the GFP transformants of *F*. *graminearum* to track its initial infection and spread [20], and Murillo et al. (1999) investigated *F*. *moniliforme* infected maize seedling in histology [21]. To precisely monitor the progress of *F*. *verticillioides* infection, transformed pathogens containing the enhanced green fluorescent protein (*EGFP*) marker gene were used in this study. Yu et al. (2017) successfully transformed the *EGFP* gene in *Fusarium* (eGFP-Tag) and use it to follow the infection process of maize stalks in northeast China [22].

There are few studies on the relationship between the infection cycle in stalk rot and ear rot, so this study aimed to clarify the infection cycle of these diseases in maize. To do this, the *EGFP* gene was expressed in the *F*. *verticillioides* strains Fv-s1 and Fv-e1 obtained from stalk and ear rot respectively, using the PEG-CaCl_2_-mediated transformation method [23] as described by Yelton et al. (1984), and three transformants of each strain were selected for subsequent experimentation, and pathogenicity assays of wild strains and transformants were conducted to test differences between the strains. An inoculation test was conducted on maize seedlings using the toothpick method [24] and the transformed isolates Fv-eGFPs1 and Fv-eGFPe1 were inoculated on different maize seeds and then hyphal colonization and degree of infection in the maize plants were confirmed with a stereo fluorescence microscope and by subsequent molecular detection.

## Materials and methods

### Ethics statement

Samples in this study were obtained from public areas that no specific permissions were required for these locations. Field sites are public access and *F*. *verticillioides* is not endangered or protected species.

### Plant material

The maize variety used in this assay was ‘Xianyu 335’, a line that is susceptible to both stalk and ear rot [25]. Plants were grown in a greenhouse maintained at 26°C with a photoperiod of 16 h created using a fluorescent lamp.

### *Fusarium* strains and plasmids

Stalk and ear rot samples were collected from 42 locations in northeastern China during 2013 and 2014. In total, 29 stalk isolates of *F*. *verticillioides* (18.6%) and 60 ear isolates (42.6%) were obtained [26]. Pathogenicity assay was conducted of all the *F*. *verticillioides* strains, that evaluated by assessing the degree of decay in the seedling radicles and coleoptiles. To further investigate the degree of infection in maize, the most pathogenic isolates from each disease Fv-s (GenBank accession number MF803750) from stalk rot and Fv-e (GenBank accession number MF803751) from ear rot, were selected.

**Fig 1:**
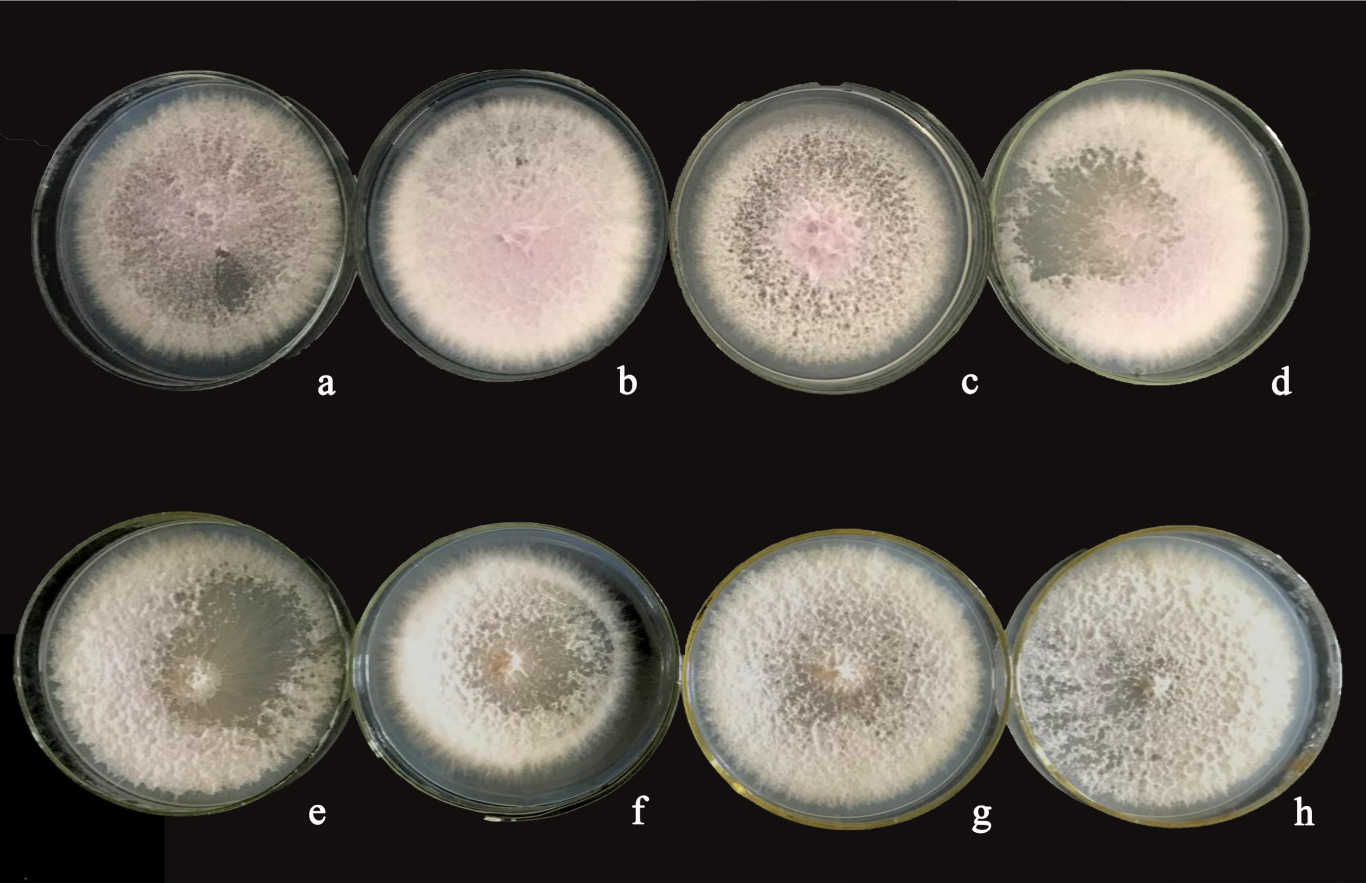
Observation of *Fusarium verticillioides* wild strains and their enhanced green fluorescent protein *(EGFP)* transformants. Colony growth (5-day-culture) of each strain. (a) stalk rot wild strain Fv-s, (b) Fv-eGFPs1, an *EGFP* transformant of Fv-s, (c) Fv-eGFPs2, an *EGFP* transformant of Fv-s, (d) Fv-eGFPs3, an *EGFP* transformant of Fv-s, (e) ear rot wild strain Fv-e, (f) Fv-eGFPe1, an *EGFP* transformant of Fv-e, (g) Fv-eGFPe2, an *EGFP* transformant of Fv-e, (h) Fv-eGFPe3, an *EGFP* transformant of Fv-e.

The plasmid pBARGPE1-Hygro-EGFP was modified from the original plasmid pBARGPE1 [obtained from Miaoling Biotechnology Company (Wuhan, China; http://www.miaolingbio.com)] to contain the *EGFP* gene. The fragment containing the *EGFP* gene, isolated from pBARKS1-egfp [27], was ligated to the pBARGPE1 vector, which is regulated by the *Aspergillus nidulans* gpdA promoter (PgpdA) and trpC terminator (TtrpC), and the bar coding region of the gene was replaced by HygB, which is controlled by the TrpC promoter. The *EGFP* gene was then transformed into *F*. *verticillioides* using the PEG-CaCl_2_-mediated transformation method [28] that according to Wang et al. (2013) [29]. The transformants that showed stable resistance against hygromycin B and fluoresced after continuous culturing for five generations were selected. Finally, three transformed strains were selected for each disease. For stalk rot the strains were Fv-eGFPs1, Fv-eGFPs2, and Fv-eGFPs3, and for ear rot, they were Fv-eGFPe1, Fv-eGFPe2, and Fv-eGFPe3 (Fig 1).

### Pathogenicity assays between wild strains and transformants

Pathogenicity assays between wild strains and their transformants were performed using corn seedlings following the methods of Gai et al. (2017) [26] in which the seedlings were evaluated by assessing the degree of decay in the radicles. Plants were placed in an incubator maintained at 26°C, and negative controls were conducted by inoculating plants with sterile inocula. The experiment was repeated twice with three replicates for each treatment.

The disease severity index (DSI) was calculated using the Townsend-Heuberger formula [30], and then subjected to an analysis of variance (ANOVA) to evaluate differences between strains. To determine which groups were statistically different, a least significant difference test (LSD; significance designated at P < 0.05) was performed using the program SPSS v16 (IBM Co., North Castle, NY, USA).

### Inoculation test

The transformed isolates Fv-eGFPs1 and Fv-eGFPe1 were used in an assay to observe the degree of hyphae infection degree throughout maize plant growth. The inoculation test was conducted in a greenhouse at Shenyang Agriculture University in May 2016. Corn kernels was loaded into plastic bags and inoculated with strain after sterilized by an autoclaving, then cultured 30 days when the mycelia overgrow with the corn kernels. Inocula were grown to the seedling stage following the method [27] of Sun et al. (2012), with slight modifications. Twenty grams of inoculum was added to the center of a pot (40 cm in diameter), and then five maize seeds were placed approximately 3 cm apart around the inoculum and covered with 3 cm of sterilized soil. A negative control was set up by inoculating seeds with sterilized inoculum. After germination, three plants were left in each pot, and the other seedlings that sprouted were removed. All the material mentioned in the assay was sterilized before using.

Sufficient water was provided once a week throughout the growing season.

A slightly modified version of the toothpick method [24] was used to inoculate adult plants. Toothpicks were boiled for 30 minutes, sterilized in an autoclave, dried, and inserted into the cultures of each strain, which had been growing on PDA (potato dextrose agar) at 26°C for 5 days. Each plant was then inoculated by inserting three toothpicks into the stem 5 cm above the soil.

In all, 200 plants, divided into two groups of 100 each (group 1 was inoculated with Fv-eGFPs1 and group 2 with Fv-eGFPe1), were tested. We collected maize plants at two points in time. The first collection comprised 20 plants from each group after 3 months, and the second, comprised the remaining 80 plants from each group when the maize was fully grown.

To demonstrate the infection cycle, the fluorescent kernels collected from the field assay were cultured on the PDA medium at 28°C for 5 days, 1×10^7^ spore suspension with maize seedlings soaked, and sterile water as control.

### Microscopic examination

Infected tissues were split lengthwise and observed for fluorescence with a stereo fluorescence microscope (Leica MZ 16F; Leica, Germany) under blue light with a 450–490 nm excitation filter and 500–550 nm emission filter. Fluorescent hyphae were viewed with a Nikon Eclipse 80i fluorescence microscope (Nikon, Japan) using an FITC (465–495 nm excitation and 510–555 nm emission) filter set and photographed with a Nikon DS-Fi1c camera and NIS-Elements software.

### Molecular detection

To determine whether the infected parts of the plants were infected with *F*. *verticillioides* strains transformed by the *EGFP* gene, a PCR was performed. The fluorescent tissue was cut and cultured on PDA medium, and then 7-day-old fungal mycelia were freeze-dried and ground into powder for genomic DNA extraction using the MiniBEST Plant Genomic DNA Extraction Kit (Takara Bio, Inc., Kusatsu, Japan).

**Fig 2:**
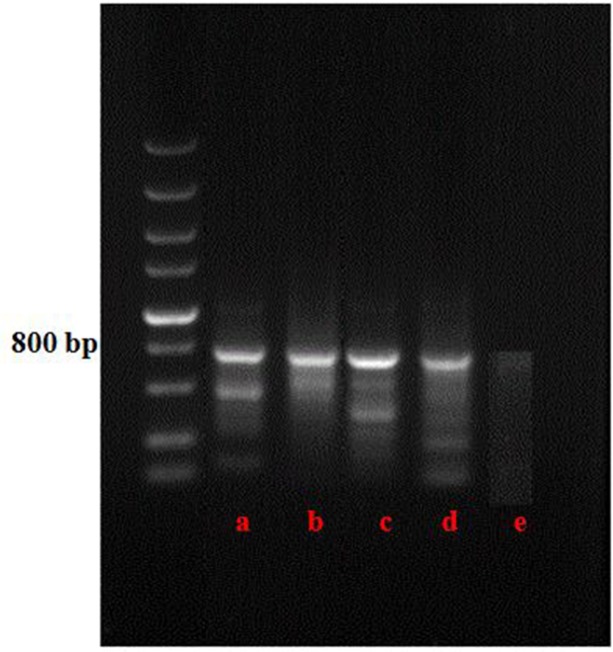
Detection of target EGFP gene of the strains by PCR amplification. a,b,c,d) were the transformants from different maize tissues, and e) was the wild type as negative control.

The target *EGFP* gene was examined with the primers egfp-f 5′-AGT GCT TCA GCC GCT AC-3′ and egfp-r 5′-CGT CCT CCT TGA AGT CG-3′ (Nai et al. 2015) [28]. The PCR program included an initial denaturation at 95°C for 5 min; 30 cycles at 94°C for 30 s, 50°C for 30s, and 72°C for 2 min; and a final extension step at 72°C for 10 min. The amplified fragments were analyzed using electrophoresis on a 0.8% (w/v) agarose gel in 1× TBE buffer (Fig 2).

## Results

### Pathogenicity assays between wild strains and transformants

The degree of decay in seedling radicles showed no significant differences in the DSI (disease severity index) values between wild strains and their transformants, although the transformants had slightly weaker virulence compared to the original wild strain (Table 1).

**Table 1:** The disease severity index of pathogenicity test between wild-type strains and transformants.

Strain	Disease severity index			Disease severity index
	Replicate 1	Replicate 2	Replicate 3	Mean (P<0.05)
Fv-s	20.0	33.3	50.0	34.4a
Fv-eGFPs1	40.0	30.0	26.7	32.2a
Fv-eGFPs2	20.0	30.0	20.0	23.3a
Fv-eGFPs3	20.0	30.0	50.0	33.3a
Fv-e	40.0	40.0	30.0	36.7a
Fv-eGFPe1	40.0	40.0	26.7	35.6a
Fv-eGFPe2	20.0	20.0	30.0	23.3a
Fv-eGFPe3	30.0	30.0	20.0	26.7a

### Degree of infection

**Fig 3:**
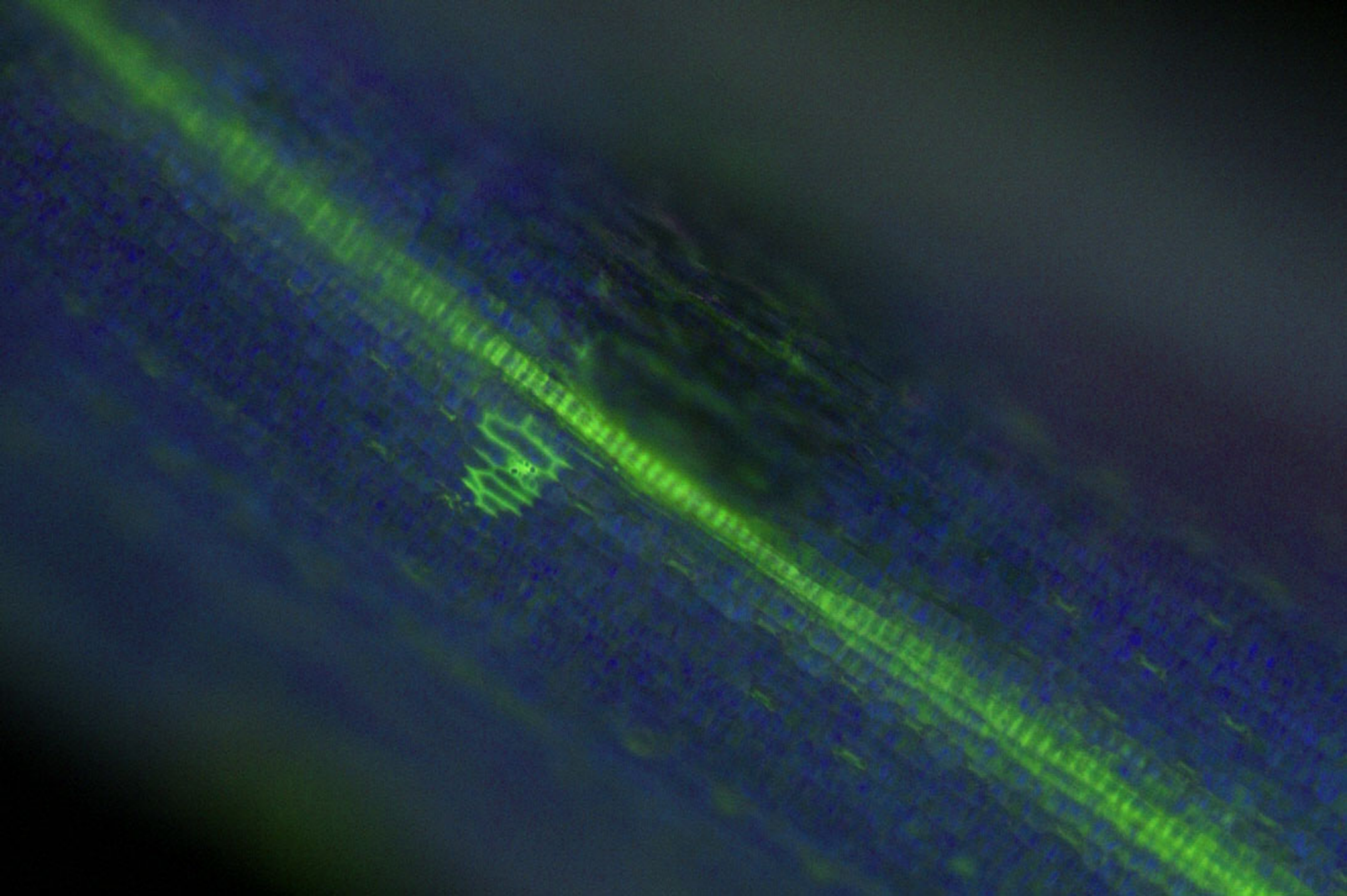
Hyphae of *Fusarium verticillioides* transformant spread the infected across cells with a rectangular extension.

At the initial seed germination stage, *F*. *verticillioides* hyphae were typically attached to the maize radicle. Some hyphae were observed penetrating the radicle into the cells, and these hyphae spread the infection to other cells with a rectangular extension (Fig 3). Next, the mycelia spread throughout the tissues infecting the rest of the plants continuously.

At the adult stage, inoculated plants were cut lengthwise revealing that most plants were infected from the first to the second stalk internode above the ground and that infected piths had turned brown and emitted green fluorescence under the stereo fluorescence microscope. Only three plants had observable green fluorescence at the third internode, and no fluorescence appeared above the third stalk internode in any plant.

**Fig 4:**
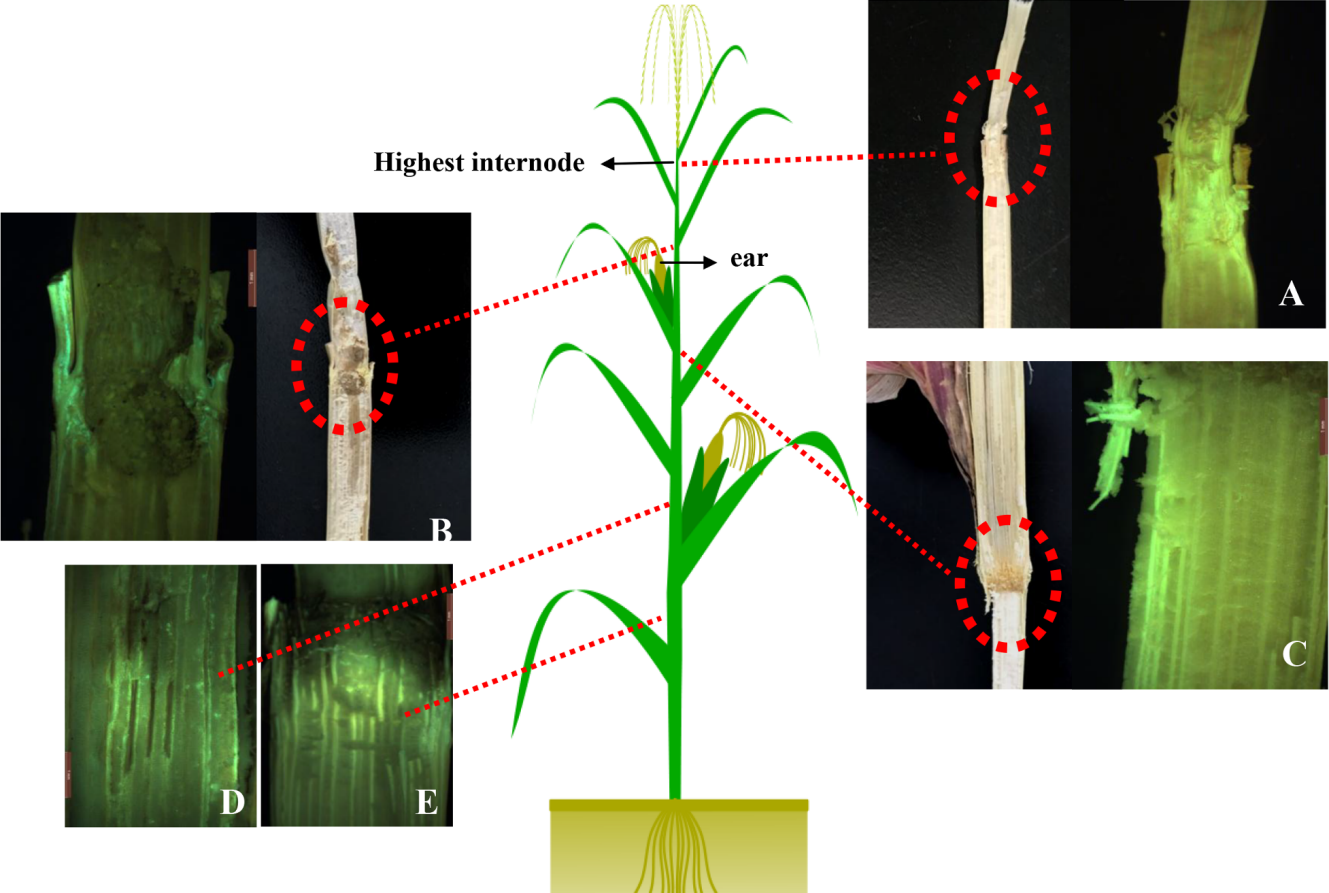
Different degrees of enhanced green fluorescent protein *(EGFP)*-tagged *Fusarium verticillioides* infection in stalks of maize. A) The highest infected internode under the natural light and blue light (Fv-eGFPe1); B) the internode above the ear under the natural light and blue light (Fv-eGFPs1); C) the internode below the ear under the natural light and blue light (Fv-eGFPe1); D and E) the below infected internode under blue light (Fv-eGFPs1).

**Fig 5:**
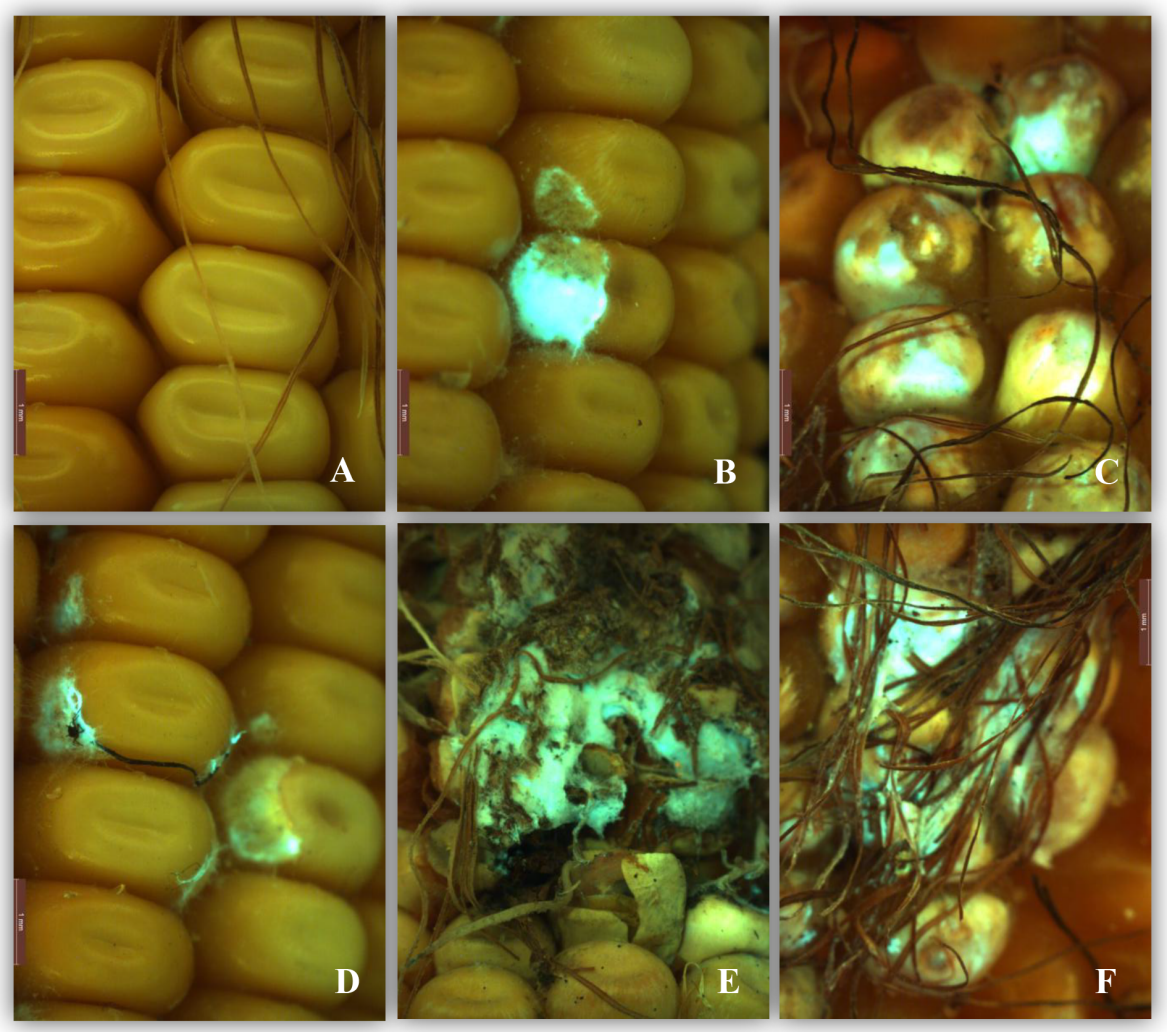
Maize ears infected with *EGFP*-tagged *Fusarium verticillioides* under blue light. A) a healthy kernel; B and C), ears infected by Fv-eGFPs1; D, E and F) ears infected by Fv-eGFPe1. Bars indicate 1 mm.

Fully ripened plants showed various degrees of infection throughout the stalk. More than half of these plants had discolored piths and fluoresced green under the stereo fluorescence microscope from the fourth to fifth stalk internode aboveground. Further, 13 plants (group 1 n = 5; group 2 n = 8) had detectable green in the tissue at higher internodes (Fig 4), and nine maize ears fluoresced (group 1 n = 3; group 2 n = 6; Fig 5). We found that necrosed piths were almost non-fluorescent, but we typically observed stronger green fluorescence above 3 cm approximately in the affected stem. It appeared that the normal pith may have already been infected by the pathogen, but not all of the infected tissue could be seen visually. Moreover, we frequently observed green fluorescence in stalks that were damaged by insects.

### Infection cycle pattern

**Fig 6:**
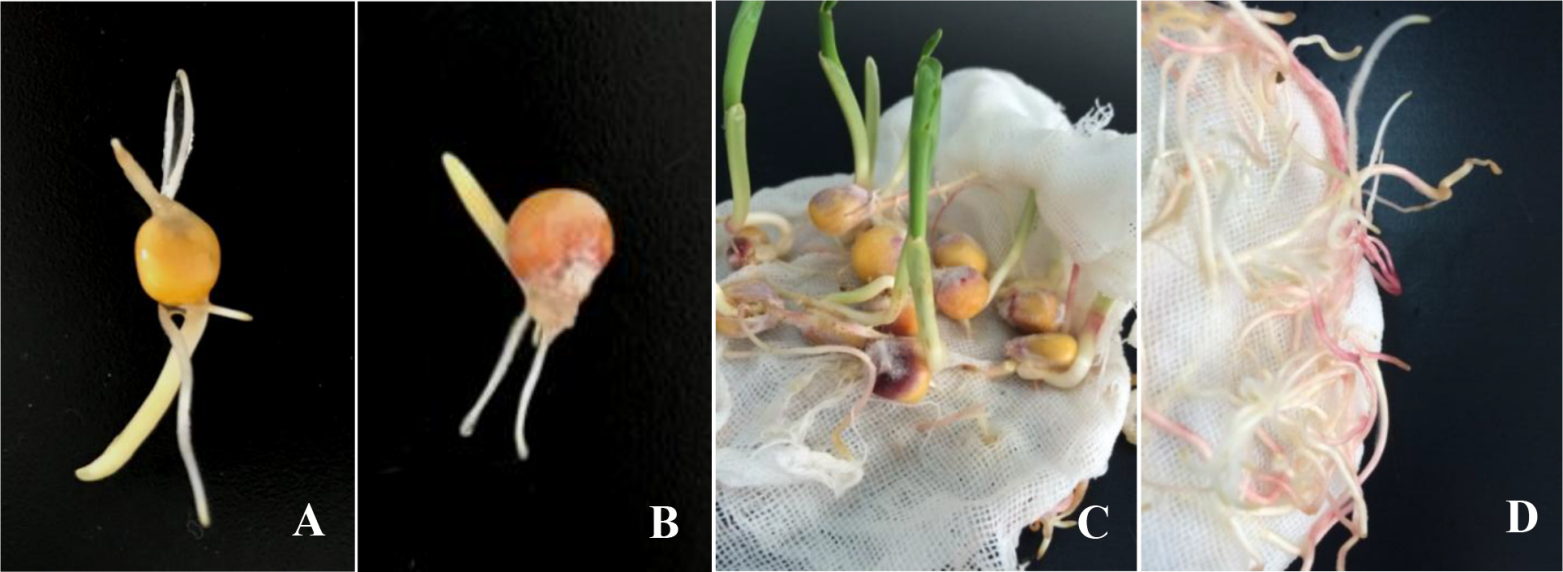
Infection cycle of maize seeds inoculated with *Fusarium verticillioides* strains cultured from fluorescent kernels. A) a healthy maize seed (negative control); B) a seed infected with the re-separated strain of fluorescent kernels; C) discolored seedlings (5-day-growth) with the re-separated strain of fluorescent kernels; D) decayed and discolor radical (5-day-growth) infected with the re-separated strain of fluorescent kernels.

Discolored seedlings and radicles exhibited decay symptoms after 24h, which the parts of symptoms became increasingly over time (Fig 6), and the seedlings as negative control shown normal growth without decay or discolored. In the test, both Fv-eGFPs1 and Fv-eGFPe1, which were obtained from stalk rot and ear rot, respectively, can infect the maize kernels that cause a second infection, and formed a infection cycle.

## Discussion

We found that all transformants had slightly weaker virulence compared to the original wild strains, but these differences were not significantly. This may have been caused by the continuous culturing on PDA, leading to a slightly reduction in the pathogenicity of the strains.

Regarding the degree of infection, more than half of the plants had observable green fluorescence from the fourth to fifth stalk internodes above the ground, and green fluorescence was detected in more plants infected with ear rot than in those infected with stalk rot, which means that there was a higher incidence of ear rot pathogens of stalk rot. This interpretation is corroborated by the occurrence of fluorescence at the highest internode. Additionally, we observed fluorescence in some stalks that lacked rot symptoms altogether. This result might have been caused by passive movement (eg. transpiration) which pulled the pathogen through the tissue. A mechanism for this type of movement has also been suggested by Duncan and Howard (2009) [31].

Furthermore, we found that insect damage influenced the severity of ear rot: the high incidence of insect damage often led to severe ear rot on maize [32]. Therefore, to prevent disease and control ear rot, pest control needs to be enhanced.

Some scholars had reported *Fusarium* from stalk rot and ear rot that exists a relationship on etiology between these two diseases [33–35]. The same *Fusarium* species from these two diseases showed high homology and was able to cross-infect.

From the assay of inoculated maize seeds, it can be concluded that *F*. *verticillioides* obtained from both diseases (stalk rot and ear rot on maize) can infect maize seeds and extend to the kernel, causing systematic infection, and the infected kernels are also pathogenetic to maize, which occurs a infection cycle from seeds to stem to ear and to seeds again.

To our knowledge, this study is the first to compare how the two pathogens from stalk rot and ear rot differed by inoculating seeds with each pathogen and investigating the infection cycle. Our results indicated that *F*. *verticillioides* obtained from either stalk rot or ear rot on maize can infect maize plants by gaining access to the plant through the radicle and then infecting the stalk upward through various tissues to the ear. Further, the infected kernels can cause a second infection when they become seeds thus completing the infection cycle. The results of the present work defined the infection cycle of *F*. *verticillioides* obtained from stalk rot and ear rot and clarified the relationship between these two diseases. Additionally, our results provide a basis for assessing the resistance of stalk rot and ear rot disease in the analyzed maize variety.
